# Image Inpainting Methods Evaluation and Improvement

**DOI:** 10.1155/2014/937845

**Published:** 2014-07-17

**Authors:** Raluca Vreja, Remus Brad

**Affiliations:** Computer Science Department, Lucian Blaga University of Sibiu, B-dul Victoriei 10, 550024 Sibiu, Romania

## Abstract

With the upgrowing of digital processing of images and film archiving, the need for assisted or unsupervised restoration required the development of a series of methods and techniques. Among them, image inpainting is maybe the most impressive and useful. Based on partial derivative equations or texture synthesis, many other hybrid techniques have been proposed recently. The need for an analytical comparison, beside the visual one, urged us to perform the studies shown in the present paper. Starting with an overview of the domain, an evaluation of the five methods was performed using a common benchmark and measuring the PSNR. Conclusions regarding the performance of the investigated algorithms have been presented, categorizing them in function of the restored image structure. Based on these experiments, we have proposed an adaptation of Oliveira's and Hadhoud's algorithms, which are performing well on images with natural defects.

## 1. Introduction

The process of region filling following the loss of information in digital images represents an important aspect in image processing. Image inpainting refers to restoration methods used to remove damage or unwanted objects from an image, in a natural manner, such that a neutral observer would not notice any changes and consider the result as being the original image.

Restoration methods can be classified in three major categories: structural inpainting techniques, textural inpainting methods, and hybrid methods. In spite of these three categories, methods may be divided in partial derivative equations (PDE) based algorithms, semiautomatic inpainting methods, texture synthesis methods, algorithms based on models/templates, and hybrid techniques depending on specific characteristics [1–3].

Based on the PDE model, the first approach belongs to Bertalmio et al. [4], who proposed a method in which the information is propagated in the occluded area, through isophote lines that cross the edges. The algorithm is efficient when applied to images with narrow damages, since it makes use of anisotropic diffusion which leads to blurring effects. The major disadvantage of this method is represented by the fact that it cannot reconstruct textures [5]. In the same category fall the methods developed by Täschler [2]. The authors have proposed an algorithm based on partial differential equations of second order which uses diffusion and an improved version of the previous one [6]. The significant problem was the same; namely, the algorithm was not able to reconstruct textures. Tschumperlé and Deriche [7] presented a method which makes use of high-order partial differential equations. Although this was not intended to be an image restoration technique, it leads to good results for images with narrow damages and occluded regions of small area.

Regarding the category of semiautomatic inpainting methods, Sun et al. [8] proposed a technique that requires two steps to perform the restoration. In the first step, the user has to sketch the object contours in the occluded area, starting from the outside to the inside, and then apply a texture synthesis process that uses images or blocks of pixels as a source for the texture. The algorithm proposed by Oliviera et al. [9] uses an isotropic diffusion process, aimed to preserve the contours. The edges are excluded from the mask in order not be affected by smoothing. Due to the iterative process, some blurring effects may be obtained. Telea [10] tries to provide an improvement, by estimating the pixel value, based on the restored pixel neighborhood, with the clear advantage of applying the inpainting process only once for each pixel, comparative to iterative methods. A seam carving method was presented in [11], overcoming the time consuming disadvantage of this type of inpainting techniques.

In the case of texture synthesis methods, the technique developed by Efros and Leung [12] uses one pixel as a starting point, located on the edge of the occluded area, defining a window around it, in order to find similar blocks in the region. This method restores texture pixel by pixel; therefore, the proposed algorithm overcomes the limitations of Bertalmio's algorithm and the similar ones.

Efros and Freeman [13] present an approach in which texture synthesis is performed using blocks, not pixel by pixel, which significantly reduces the execution time. The algorithm has proven to be more efficient by copying an entire block when a valid candidate is found in the source. Although the method is much faster and therefore more efficient, yet it fails to provide good results for images with highly structured textures.

Heeger and Bergen [14] proposed a texture reconstruction method using a collection containing intermediate images that form a so-called image pyramid. Their method consists of an iterative process in which the image pyramid is created by dividing the damaged image and the one representing the source. According to the authors, repeating the process for a number of steps, a texture with satisfactory results will be obtained, yet valid only for stochastic types. In the paper of de Bonet [15], an improvement was proposed in order to reproduce also regular textures. This is achieved by taking into account dependencies between different levels of texture granularity. Igehy and Pereira [16] describe another version of the algorithm proposed by Heeger and Bergen, involving a new step that uses a mask containing subunit values, aiming to specify the amount of information from the original image used for synthesizing the texture.

The same inpainting category could include an algorithm based on templates, developed by Criminisi et al. [17]. The authors are describing a technique highlighting the importance of the order in which pixels are restored. The algorithm starts from the edge of the occluded area, assigning each pixel from the edge a priority. Texture synthesis is done with blocks, by replicating information from a source area, depending on the priority value determined for each pixel.

The algorithm proposed by Drori et al. [2, 18] focuses on the details of granularity levels, which are used as an estimation of the best levels. It then sets a filling order by means of a confidence value, followed by a search step similar to Efros and Leung. Their algorithm uses several different orientations of the block. The inpainting algorithm of Guillemot et al. [19] searches the k-nearest neighbors of the damage to be filled and linearly combines them in order to replace the restored pixels. The k-nearest neighbor search is then improved by linear regression.

Hays and Efros [20] present a method that uses a large image collection as a database for restoration. The authors point out that the possibility to restore the region in a natural manner increases due to the amount of information contained in the large images set. The restoration process is done by checking each item in the database for a possible match of the damaged region using an image descriptor. The same approach was presented by Le Meur and Guillemot [21], introducing an exemplar-based inpainting framework. A coarse version is first inpainted, allowing reducing the computational complexity and noise sensitivity and extracting the dominant orientations of image structures. A novel concept of sparsity at the patch level is proposed by Xu and Sun [22], in order to model patch priority and patch representation, two important steps for patch propagation in exemplar-based inpainting. Aujol et al. [23] provide experimental confirmation of the fact that exemplar-based algorithms could reconstruct local geometric information, while the minimization of variational models allows a global reconstruction of geometry and especially of smooth edges.

One of the hybrid inpainting methods belongs to Bertalmio et al. [24], who developed an algorithm based on the idea of decomposing the original image in two layers. One layer should contain the structural characteristics and the second the texture. The first image would be processed by a structural inpainting algorithm [4] and the second one would be processed by the texture synthesis algorithm proposed by Efros and Leung in [12]. The results of both operations contribute to the final image. Another hybrid method was proposed by Atzori and de Natale [25]. In this case, the restoration process starts from matching the contours that crosses the edge of the occluded area in its interior. This operation will lead to smaller regions that will be filled by copying blocks from the outside. Rareş et al. [26] proposed that both the local and the global information should be taken into consideration for the union of the edge contours intersecting the damaged area. Thus, pairs of lines which are more accurate will be obtained, but the matching process will be more complicated. Restored pixel values are then assigned according to the pixels in the proximity of the new contours obtained and according to the edges of the occluded area.

## 2. The Inpainting Techniques Used in Our Evaluation

For the scope of our research, five inpainting algorithms were chosen. The first algorithm was developed by Bertalmio et al. [4] and represents a reference inpainting method. The second, presented in [9], depicts a simple solution, based on a convolution operation, followed by the third, an adapted version of the previous [27]. The following technique was proposed by Efros and Leung [12] for texture synthesis and the last considered algorithm from Criminisi et al. [17], combining techniques for structured inpainting and texture reproduction.

### 2.1. Bertalmio's Algorithm

In order to obtain the restored image, it is necessary to interleave inpainting steps with a number of anisotropic diffusion steps. Considering the occluded area Ω and the contour of the region ∂Ω, the purpose of the method is to propagate the information along isophote lines that crosses the contour *∂*Ω [4]. The algorithm operates iteratively and creates a family of images, each image representing an improved version of the previous one. Consider

(1) $$\begin{array}{c} I^{n+1}\left(i,j\right)=I^{n}\left(i,j\right)+\Delta tI_{t}^{n}\left(i,j\right),\;\forall \left(i,j\right)\in \Omega , \end{array}$$

where *I* ^*n*+1^(*i*, *j*) is the intensity of the pixel having the coordinates (*i*, *j*) at moment *n* + 1, Δ*t* is the improvement or change rate, and *I*_*t*_^*n*^(*i*, *j*) corresponds to an image update at time *n*. This update includes the information to be propagated and the direction of propagation, as follows:

(2) $$\begin{array}{c} I_{t}^{n}\left(i,j\right)=\left(\vec{\delta L^{n}}\left(i,j\right)\cdot \frac{\vec{N}\left(i,j,n\right)}{\left|\vec{N}\left(i,j,n\right)\right|}\right)\left|\nabla I^{n}\left(i,j\right)\right|, \end{array}$$

where $\vec{\delta L^{n}}$ is a vector indicating the intensity change in the image, obtained after applying the Laplace operator. The isophote line direction is expressed as follows:

(3) $$\begin{array}{c} \frac{\vec{N}\left(i,j,n\right)}{\left|\vec{N}\left(i,j,n\right)\right|}=\frac{\left(-I_{y}^{n}\left(i,j\right),I_{x}^{n}\left(i,j\right)\right)}{\sqrt{{\left(I_{x}^{n}\left(i,j\right)\right)}^{2}+{\left(I_{y}^{n}\left(i,j\right)\right)}^{2}+\varepsilon }}, \end{array}$$

where *ε* is a small value intended to avoid potential division by 0 and *I*_*x*_^*n*^, *I*_*y*_^*n*^ are intensities determined by the difference between the intensities of the next pixel and the previous one. The slope, limited norm of the gradient, has the aim of improving the stability:

(4) |∇In(i,j)|={(Ixbmn)2+(IxfMn)2+(Iybmn)2+(IyfMn)2, βn>0,(IxbMn)2+(Ixfmn)2+(IybMn)2+(Iyfmn)2, βn<0,βn(i,j)=δLn→(i,j)·N→(i,j,n)|N→(i,j,n)|.

Indices *b* and *f* specify the difference between the intensities of the current pixel and the one in the reverse direction or forward, on* OX* and* OY* coordinate axes. Indices *m* and *M* express the fact that the minimum or the maximum value between the obtained result and 0 will be chosen.

The method proposed by Bertalmio et al. interleaves a number of *A* inpainting steps with *B* anisotropic diffusion steps, where* A*,* B*, and* T* (total number of iterations) are input parameters. We have used an anisotropic diffusion proposed by Perona and Malik [28], presenting a function limiting the diffusion process to homogeneous regions:

(5) $$\begin{array}{c} I_{i,j}^{t+1}=I_{i,j}^{t}+\lambda {\left[c_{N}\cdot \nabla_{N}I+c_{S}\cdot \nabla_{S}I+c_{E}\cdot \nabla_{E}I+c_{W}\cdot \nabla_{W}I\right]}_{i,j}^{t}, \end{array}$$

where *I*_*i*,*j*_^*t*+1^ is the intensity of the pixel having the (*i*, *j*) coordinates at *t* + 1 moment, *λ* is a constant value which should be in the range [0, 0.25] for algorithm stability, and ∇_*N*_ *I*,  ∇_*S*_ *I*,  ∇_*E*_ *I*,  ∇_*W*_ *I* represent the difference between the intensities of the pixel in the direction indicated by the index (north, south, east, or west) and the current pixel:

(6) $$\begin{array}{c} \nabla_{N}I_{i,j}\equiv I_{i,j-1}-I_{i,j},\\ \nabla_{S}I_{i,j}\equiv I_{i,j+1}-I_{i,j},\\ \nabla_{E}I_{i,j}\equiv I_{i+1,j}-I_{i,j},\\ \nabla_{W}I_{i,j}\equiv I_{i-1,j}-I_{i,j} \end{array}$$

with *c*_*N*_,  *c*_*S*_,  *c*_*E*_,  *c*_*W*_ called conduction coefficients, determined based on the gradient. There are several methods to compute these values, including the following two, proposed by the authors:

(7) c(||∇I||)=e−(||∇I||/K)  2,

(8) c(||∇I||)=11+(||∇I||/K)2,

The coefficients are determined using one of (7) or (8), where the gradient ∇*I* corresponding to the direction described by the index and *K* controls the sensitivity of the edge detection process. Both the inpainting stage itself and the anisotropic diffusion method will be applied to the RGB components of the pixel.

### 2.2. Oliveira's Algorithm

**Figure 1:**
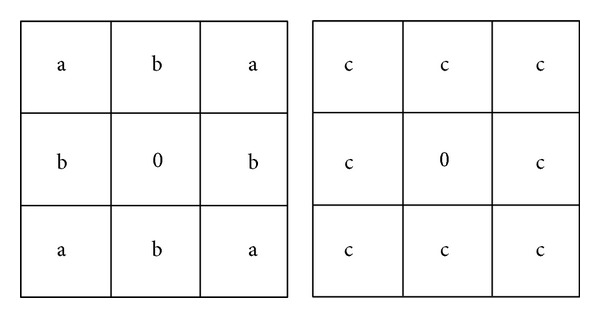
The convolution kernels proposed by Oliviera et al. [9].

Based on the previous method, Oliviera et al. [9] have proposed an inpainting algorithm that relies exclusively on diffusion. The processing steps consist of deleting color information inside the mask followed by edge detection for the occluded area. Starting from the pixels on the edge, a convolution operation is then applied, using a neighborhood centered on each contour pixel and one of the kernels proposed (Figure 1). The values of* a*,* b,* and *c* for both kernels are 0.073235, 0.176765, and 0.125, respectively [9].

### 2.3. Hadhoud, Moustafa, and Shenoda's Algorithm

**Figure 2:**
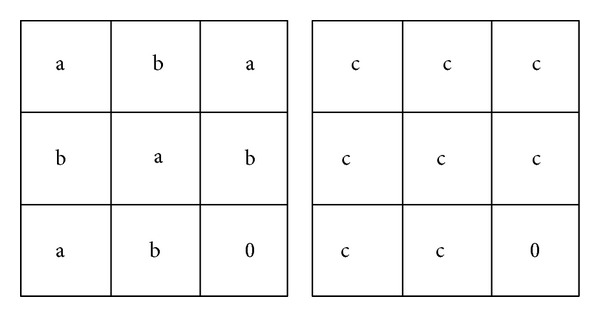
The convolution kernels proposed by Hadhoud et al. [27].

Hadhoud et al. [27] have proposed an improvement of Oliveira's method, regarding both the final image and the required processing time. Some steps have been kept from the original method of [9], involving the selection of the mask, followed by the removal of the existing color information in the mask. Unlike Oliveira's algorithm, the method uses a differently defined convolution kernel. The idea was to use as much as possible information from outside of the region, in view of the restoration process (Figure 2). By using more known neighbors, the restoration can be achieved even within a single iteration.

### 2.4. Efros and Leung's Algorithm

The algorithm steps include defining a mask and specifying a source area, followed by the edge detection for the occluded area [12]. All pixels on the edge will be sorted in descending order by the number of known neighbors. A template will be defined centered for each pixel chosen for restoration. This window has a parameterized size and it will be used in searching for similar blocks in the source area. The similarity measure is given by the sum of squared differences (SSD). To preserve the local character of the texture, a Gaussian kernel is used, which aims to control the influence of pixels located too far from the occluded area. Consider

(9) $$\begin{array}{c} d=d_{\text{SSD}}*G. \end{array}$$

Depending on the SSD value, a collection of candidate blocks will be obtained. Consider

(10) $$\begin{array}{c} \omega_{\text{best}}=\underset{\omega }{\arg\;\min}d\left(\omega \left(p\right),\omega \right)\subset I_{\text{smp}},\\ d\left(\omega \left(p\right),\omega \right)<\left(1+\varepsilon \right)d\left(\omega \left(p\right),\omega_{\text{best}}\right),\;\varepsilon =0.1, \end{array}$$

where the processed pixel is *p*, *I*_smp_ represents the source area, and *d*(*ω*(*p*), *ω*) describes the distance from a *ω* sized window centered on pixel *p* to a block of the same size *ω*, found in the source. One of the candidate blocks will be chosen randomly and the color information of its center pixel will be assigned to the pixel on the edge (the center of the window template).

### 2.5. Criminisi's Algorithm

This algorithm aims to achieve texture synthesis, taking into consideration structural information, such as the isophote lines that cross the edge of the occluded area [17]. It consists of three major steps and starts with the pixels on the edge ∂Ω of the mask Ω. For all windows centered on edge pixels, a priority *P*(*p*) is computed, where *p* represents the processed pixel at a certain moment:

(11) $$\begin{array}{c} P\left(p\right)=C\left(p\right)*D\left(p\right), \end{array}$$

where *C*(*p*) represents a confidence term associated with a block *ψ*_*p*_ (the higher the number of known pixels in the window, the higher the confidence). *D*(*p*) is a term that processes the structural information contained in the window *ψ*_*p*_ and raises the priority of a block comprising an isophote line. These two terms are defined as follows:

(12) C(p)=∑q∈ψp∩(I−Ω)C(q)|ψp|,D(p)=|∇Ip⊥np|α

with |*ψ*_*p*_| the surface of the window *ψ*_*p*_ centered in pixel *p* belonging to ∂Ω, where *α* is a normalization factor with value 255, *n*_*p*_ is the normal to the contour ∂Ω at point *p*, and ∇*I*_*p*_^⊥^ is the normal to the gradient, namely, the isophote line.

For the priorities *P*(*p*) an initialization step is required. All pixels belonging to the mask have the confidence term *C*(*p*) = 0 and the ones belonging to the source band have the confidence *C*(*p*) = 1.

The second processing step represented the inpainting itself. The pixel having the highest priority is the first to be processed; its associated source block from the source area is the one that leads to a minimal SSD distance:

(13) $$\begin{array}{c} \psi_{\hat{q}}=\underset{\psi_{q\in \Phi }}{\arg\;\min}\;d\left(\psi_{\hat{p}},\psi_{q}\right), \end{array}$$

where $d(\psi_{\hat{p}},\psi_{q})$ represents the SSD value (between all known pixels of the window *ψ*_*p*_ and the ones on the corresponding positions in a block *ψ*_*q*_ belonging to the source band). Knowing the source window *ψ*_*q*_, all pixels of *ψ*_*p*_ that also belong to the mask, will be filled with information provided by the corresponding pixels in *ψ*_*q*_. The last step consists of updating the confidence values associated with pixels in the restored window:

(14) $$\begin{array}{c} C\left(p\right)=C\left(\hat{p}\right),\;\forall p\in \psi_{\hat{p}}\cap \Omega . \end{array}$$

## 3. A Proposed Adaptation of Oliveira's and Hadhoud's Algorithms

Concerning the algorithm developed by Oliveira and its adaptation proposed by Hadhoud et al. [27], conserving edges is one of the major problems. Therefore, Oliveira et al. defined some diffusion barriers over the contour in order to stop the isotropic diffusion process; otherwise, some visible blurring effects may occur. However, in the case of Hadhoud et al., redefining the kernel and the direction of propagation leads to even more highlighted blurring effects and the loss of contour lines.

As an alternative to the 2-pixel width barriers defined according to Oliveira's idea, we are proposing an edge conserving procedure by defining an additional mask that comprises the contour. The mask will be processed using an anisotropic diffusion operation described in Bertalmio's algorithm. The mask pixels are excluded from the initial mask and will no longer be modified using one of the kernels of isotropic smoothing operation. As a result, the user intervention is simplified and the results are satisfactory.

Oliveira's and Hadhoud's methods are suited for images with natural defects such as Lincoln. Unfortunately, the original image (without defects) does not exist; therefore, we could not compute the PSNR in comparison to it. In order to reach a conclusion regarding these methods and our proposal for edge preserving, some images were chosen and defects were manually applied. Therefore, the PSNR could be computed by comparing the restored image with the original one.

**Figure 3:**
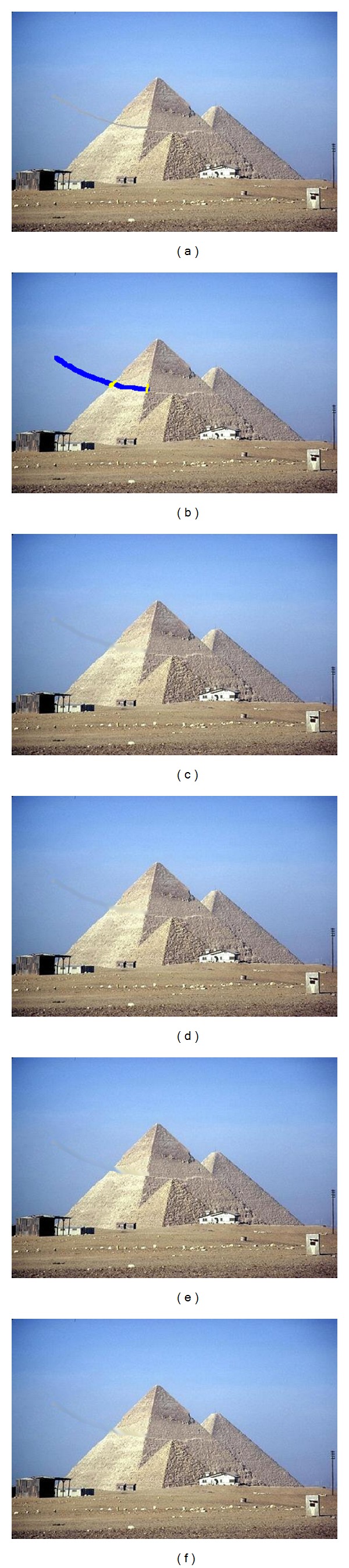
Visual comparison of the proposed methods; (a) simulation of a natural defect; (b) the corresponding masks; (c) result of the Oliveira method; (d) result of the proposed adaptation of Oliveira method; (e) result of the Hadhoud method; (f) result of the proposed adaptation of Hadhoud method.

In the image shown in Figure 3(a), we have applied a defect that could be considered close to a natural one. The blue mask will be processed using Oliveira's or Hadhoud's method, as for the yellow mask, an anisotropic diffusion will be applied. It can be noticed from the result in Figure 3 and Table 1 that our proposal offers improvements regarding Hadhoud's method. However, it worth mentioning that the results would be more relevant if images with natural defects would have been tested and their originals could be used as a ground truth.

## 4. An Evaluation of the Inpainting Algorithms

The five inpainting methods were implemented in the C# and run on a system with Intel i5 processor at 2.5 GHz. The method proposed by Bertalmio et al. was implemented on RGB color images. The algorithm developed by Oliveira et al. and the method proposed by Hadhoud et al. were implemented taking into consideration the proposal described above regarding edge conservation. In the case of Efros and Leung's algorithm, the source area was represented by a band around the occluded region [2]. The same assumption was considered for the method proposed by Criminisi et al. Our evaluation was carried out on representative test images, characterized by structural lines, but also by texture content.

**Figure 4:**
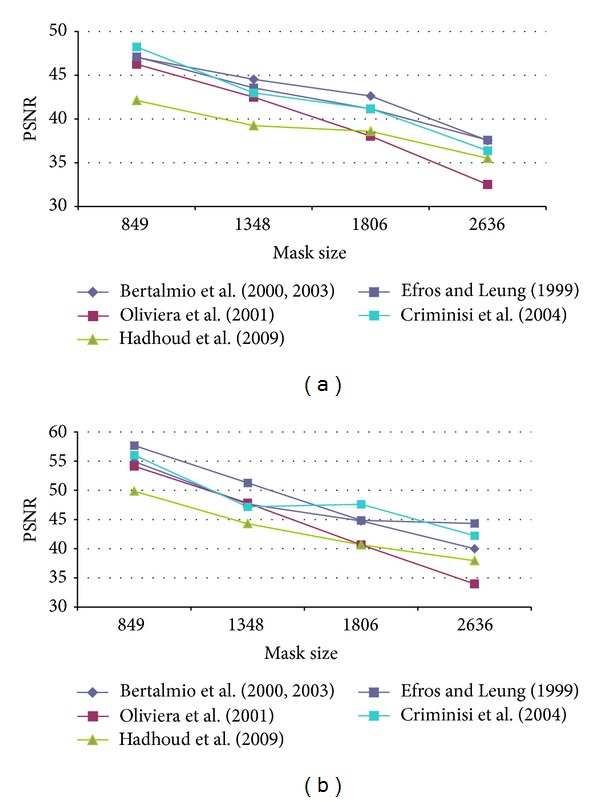
PSNR results for (a) Lena and (b) Peepers test image.

**Figure 5:**
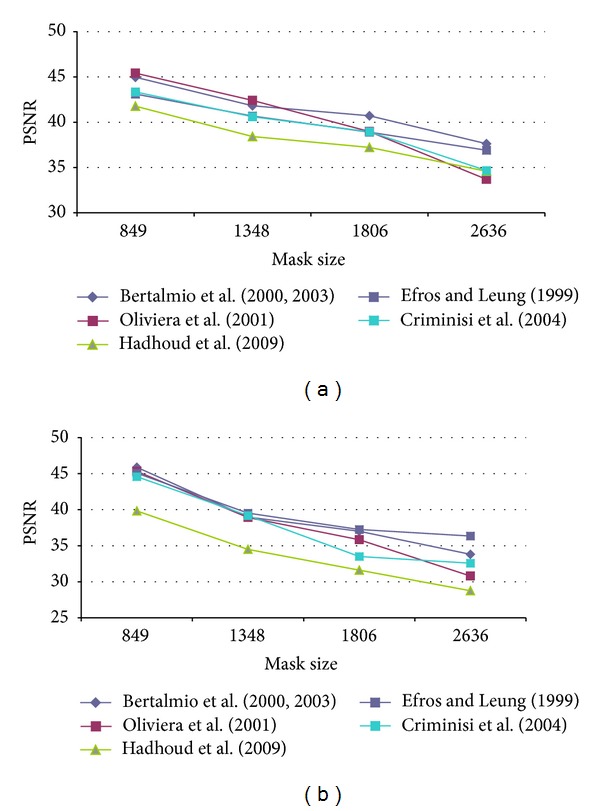
PSNR results for (a) Baboon and (b) StillLifeWithApples test image.

**Figure 6:**
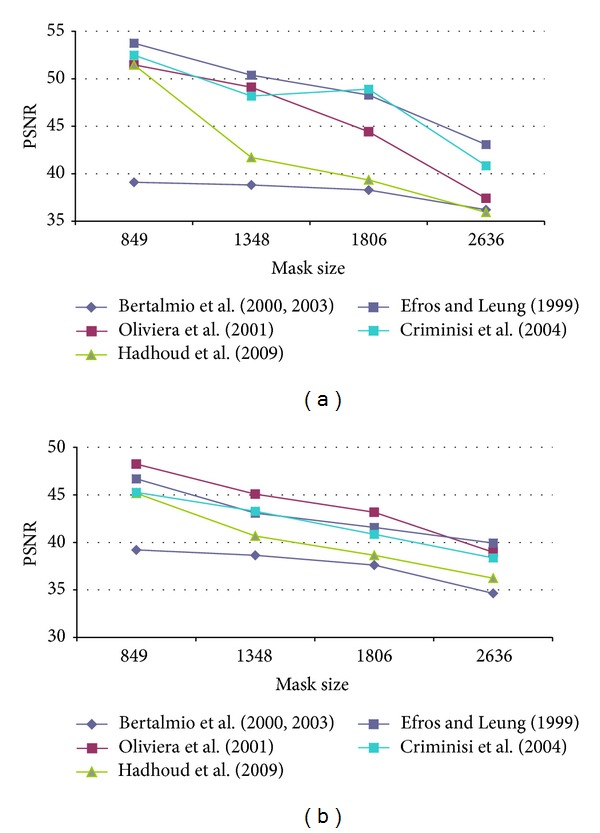
PSNR results for (a) Barbara and (b) Egipt test image.

**Figure 7:**
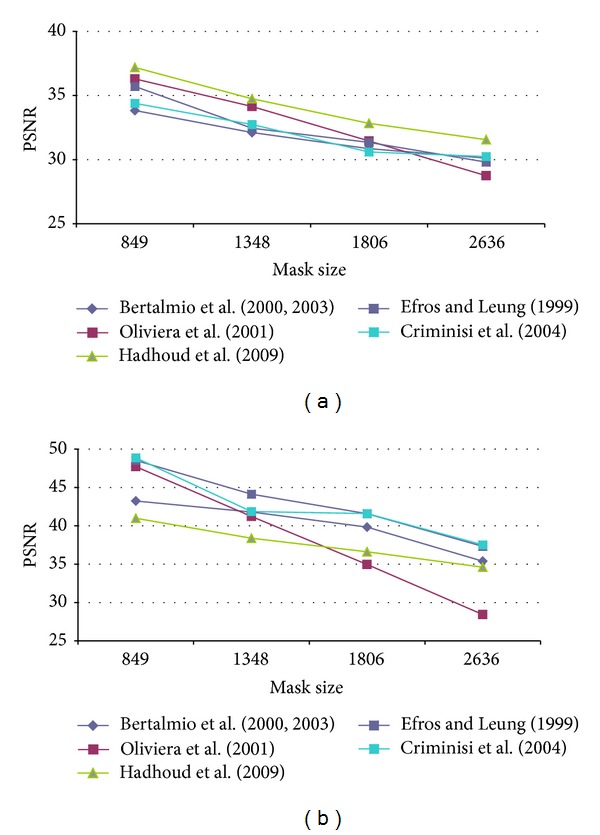
PSNR results for (a) cat fur and (b) fly test image.

**Figure 8:**
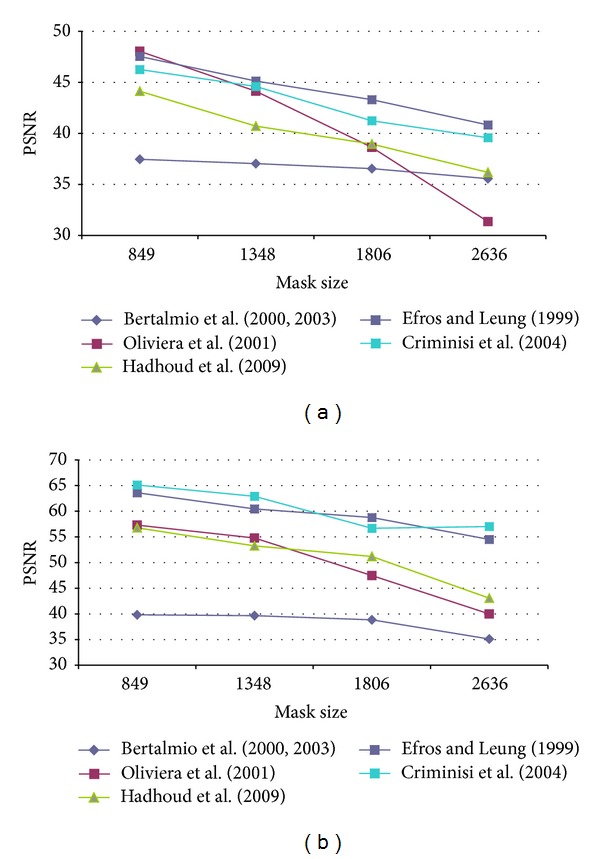
PSNR results for (a) helicopter and (b) lands test image.

First of all, it was necessary to determine the optimal configuration of each method parameters in order to obtain the best results in terms of PSNR. Therefore, several configurations for each algorithm were tested. The test images used were Lena, Peppers, Baboon, and StillLifeWithApples as presented in [17] and Barbara, Egipt, cat fur, fly, helicopter and lands from [29]. An artificial damage was applied and the restored image was compared to the original one as reference. Oliveira's method and the version proposed by Hadhoud et al. were tested on the well-known inpainting test images Lincoln and Three Girls, due to their efficiency on natural damage images. The main disadvantage was that there are no original images that could be used as reference, in order to compute the PSNR value. Our artificial test damage was defined as a stripe, successively widened, in order to notice how the algorithm behaves for “spot masks.” The data in Table 2 presents the mask (damage) size in pixels and the corresponding initial PSNR values. By gradually increasing the mask width, we had obtained the PSNR results presented in Figures 4, 5, 6, 7, and 8 for the ten considered test images.

As it can be seen from the PSNR results, among the structural inpainting methods, the one belonging to Bertalmio leads to the successful results, among which Peppers and Lena obtain the highest values. Due to diffusion method, the algorithm has lower results for textural images in comparison with structural ones.

**Figure 9:**
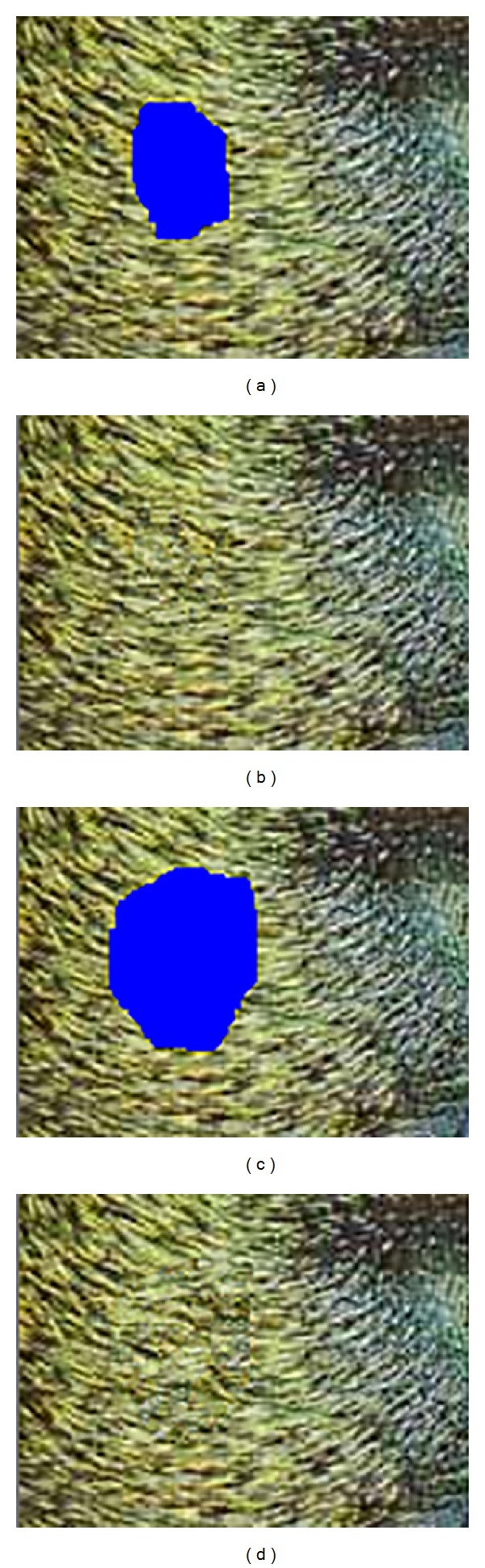
Test images with damage of (a) 983 pixels, (c) 2120 pixels and the corresponding results (b) and (d) obtained with Criminisi's algorithm.

For the last two methods, there are some improvements, but it is important to mention that, in the case of textural images, the PSNR value is not relevant, as inpainting is performed by the replication of information from a source area and not by actual propagation inside the mask. Consequently, as the mask increases, it is likely to obtain lower PSNR values and still have a very successful visual effect (as it can be seen from Figure 9). In the case of diffusion methods, the results are less successful, leading to color spread and causing blurring effects.

Considering the proposed adaptation for contour line preserving of Oliveira's and Hadhoud's methods described in Section 3, an improvement has been noticed in comparison to the basic algorithm, which applied isotropic diffusion over the entire mask. Unfortunately, since these two methods are suitable for natural defects images, they cannot be compared to an original (unaltered) image. In this case, the PSNR value would be computed in comparison with other restored images from the literature, indicating the similarity to them, and the obtained values would not be a proof of a successful restoration. There are no original images for Lincoln and Three Girls (highly referenced in the domain); therefore, a conclusive PSNR value could be determined and only a visual analysis would be possible.

**Figure 10:**
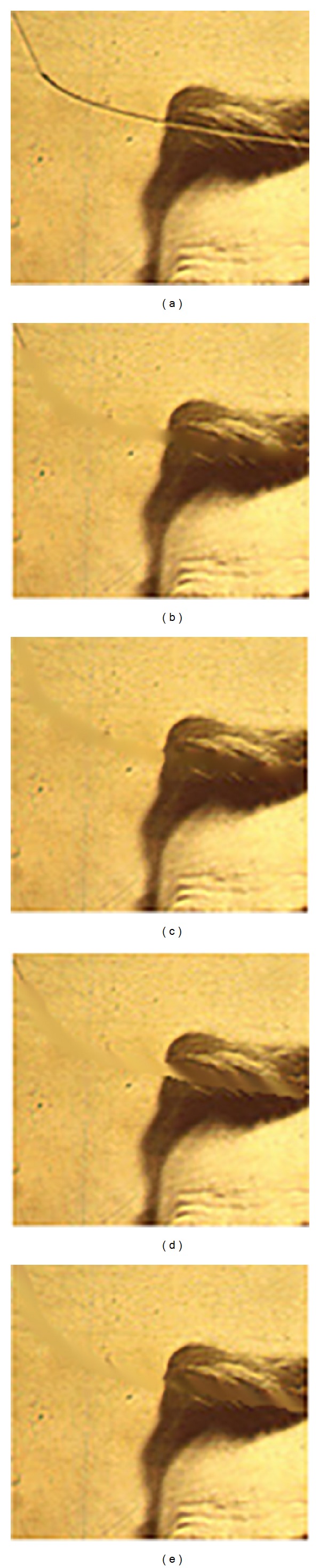
(a) Original image; (b) result for Oliveira's method; (c) result for our adaptation of Oliveira's method; (d) result for Hadhoud's method; (e) result for our adaptation of Hadhoud's method.

However, the visual restoration is satisfactory, as it can be seen from Figures 10(c) and 10(e), and is processed using our proposed method for edge preserving applied to Oliveira's and Hadhoud's methods, respectively. In comparison with the original Oliveira method, where the obtained edge was blurred (as shown in Figure 10(b)), our approach offers better contour preservation (Figure 10(c)). Also, due to the kernel used in Hadhoud's method, the edge is altered (Figure 10(d)). However, applying the proposed method in combination with Hadhoud's leads to good visual results (Figure 10(e)). We will conclude that using our new procedure, in combination with Oliveira's and Hadhoud's methods, will offer advantages in the case of natural defects images such as Lincoln and Three Girls.

It was found that the algorithm proposed by Bertalmio et al. successfully restores images, when the method is applied to reduced surface masks or with narrow width, because the contour lines crossing the area can be properly connected. The major disadvantage of the algorithm is that for large masks, due to diffusion, a blurring effect occurs and, therefore, the algorithm fails to restore textural images. The method, however, can lead to good results using small amount of information around the mask, unlike the texture inpainting algorithms, which requires a more significant amount of information in order to perform the restoration.

Unlike the algorithm proposed by Bertalmio et al., the method presented by Oliveira et al. is less complex. However, this advantage fails to compensate the fact that the contour lines can be preserved only by defining the diffusion barriers and the algorithm can be successfully applied to images with natural damage. Therefore, the algorithm is suitable for masks having narrow width; otherwise, a high blurring effect can be noticed.

In the case of Hadhoud et al. method, processing time improvements could be noticed, as a consequence of the fact that more known neighbors of the restoring pixel are used. Hence, the required number of iterations considerably decreases. Similarly to the Oliveira et al. method, the algorithm is suitable for restoring images that do not have high contrast.

The texture synthesis algorithm proposed by Efros and Leung led to impressive results. Although in contrast to other methods, the numerical values may be less satisfactory, because the stochastic textures would be impossible to restore. The restored pixels have been assigned a close value to the original one as inpainting is done by copying pixels from a predetermined area and not by propagation of external information. The method performs well also for structural images, but the main disadvantage consists of the extremely long processing time, caused by the pixel by pixel restoration.

The Criminisi method leads to good results both for structural and textural images, since it takes into consideration structural information. Unlike the Efros and Leung algorithm, restoration is performed block by block, reducing the processing time. As a consequence, a disadvantage may occur when choosing too large blocks for replication, as inappropriate information can be copied inside the occluded area. The quality of the results heavily depends on this parameter, but also on the provided context by means of a second parameter, which specifies the source bandwidth.

## 5. Conclusions

The paper presents a comparative study regarding inpainting techniques in order to evaluate different types of image restoration methods and to emphasize the advantages and disadvantages for each of the approached algorithms.

Due to the fact that a certain number of inpainting methods have been proposed during the last years, it is still difficult to designate the appropriate one. The algorithms chosen for our evaluation are representative for the categories they belong to, having as reference the first one, developed by Bertalmio. Other methods were also analyzed, as the one proposed by Oliveira and its adapted version proposed by Hadhoud et al., suitable for images without textures. Regarding these two methods, an alternative to the diffusion barriers was proposed by us. The restoration of textured images had also been taken into account in our evaluation, by using the method developed by Efros and Leung and the algorithm proposed by Criminisi.

It was also important to determine the algorithm parameters that lead to the best PSNR results and selecting representative test images to provide relevant information. The images were restored, gradually varying the width of the occluded area, in order to analyze the influence of this parameter. The tests have shown that inpainting algorithms involving diffusion operations perform well for structural features images but cannot successfully rebuild textures.

Image restoration using the RGB color system for the algorithm developed by Bertalmio led to successful results for structural images. The adaptation proposed for Oliveira's and Hadhoud's algorithms has been proven to be a successful alternative for edge preserving, with remarkable results. However, textural inpainting techniques are the most successful. Even if requiring a longer processing time, they perform well on both image types.

Further developments of this work may consist of implementing hybrid methods that combine features of the approached algorithms and comparing their results with the ones belonging to the already analyzed methods. Hybrid methods would require reconstruction processes for the contour lines and restoration processes over the obtained regions by means of textural inpainting techniques.

## Figures and Tables

**Table 1:** PSNR values comparison for the proposed methods.

Image	Oliveira	Hadhoud	Our adaptation of Oliveira	Our adaptation of Hadhoud
Peppers	47,155	42,383	46,7605	43,138
Egipt	46,3948	43,8093	46,038	46,00067

**Table 2:** The initial PSNR values depending on the mask size.

Image	Mask size (pixels)			
	849	1348	1806	2636
Lena	37.26898	34.9823	33.0214	30.23914
Peppers	34.61765	32.1941	30.4215	27.8131
Baboon	32.7125	30.1962	28.43159	25.7326
StillLifeWithApples	32.9604	30.7468	28.9399	26.3172
Barbara	33,43815	31,12731	29,333	26,6095
Egipt	29,94907	27,5565	25,72072	23,0565
Cat fur	32,10376	29,58541	27,61433	25,1814
Fly	30,935	28,547	26,70904	23,9389
Helicopter	35,3284	33,0786	31,32791	28,7093
Lands	30,3642	28,0075	26,2049	23,4996

## References

[1] Patel P, Prajapati A, Mishra S (2012). Review of different inpainting algorithms. *International Journal of Computer Applications*.

[2] Täschler ME (2006). A comparative analysis of image inpainting.

[3] Guillemot C, Le Meur O (2014). Image inpainting: overview and recent advances. *IEEE Signal Processing Magazine*.

[4] Bertalmio M, Sapiro G, Caselles V, Ballester C Image inpainting.

[5] Bugeau A, Bertalmio M, Caselles V, Sapiro G (2010). A comprehensive framework for image inpainting. *IEEE Transactions on Image Processing*.

[6] Chan TF, Shen J (2001). Nontexture inpainting by curvature-driven diffusions. *Journal of Visual Communication and Image Representation*.

[7] Tschumperlé D, Deriche R (2005). Vector-valued image regularization with PDEs: a common framework for different applications. *IEEE Transactions on Pattern Analysis and Machine Intelligence*.

[8] Sun J, Yuan L, Jia J, Shum HY (2005). Image completion with structure propagation. *ACM Transactions on Graphics*.

[9] Oliviera MM, Bowen B, McKenna R, Chang YS Fast digital image inpainting.

[10] Telea A (2004). An image inpainting technique based on the fast marching method. *Journal of Graphics Tools*.

[11] Yan B, Gao Y, Sun K, Yang B Efficient seam carving for object removal.

[12] Efros AA, Leung TK Texture synthesis by non-parametric sampling.

[13] Efros AA, Freeman WT Image quilting for texture synthesis and transfer.

[14] Heeger DJ, Bergen JR Pyramid-based texture analysis/synthesis.

[15] de Bonet JS Multiresolution sampling procedure for analysis and synthesis of texture images.

[16] Igehy H, Pereira L Image replacement through texture synthesis.

[17] Criminisi A, Pérez P, Toyama K (2004). Region filling and object removal by exemplar-based image inpainting. *IEEE Transactions on Image Processing*.

[18] Drori I, Cohen-Or D, Yeshurun H (2003). Fragment—based image completion. *ACM Transactions on Graphics*.

[19] Guillemot C, Turkan M, Meur OL, Ebdelli M Image inpainting using LLE-LDNR and linear subspace mappings.

[20] Hays J, Efros A (2007). Scene completion using millions of photographs. *ACM Transactions on Graphics (SIGGRAPH 2007)*.

[21] Le Meur O, Guillemot C Super-resolution-based inpainting.

[22] Xu Z, Sun J (2010). Image inpainting by patch propagation using patch sparsity. *IEEE Transactions on Image Processing*.

[23] Aujol J, Ladjal S, Masnou S (2010). Exemplar-based inpainting from a variational point of view. *SIAM Journal on Mathematical Analysis*.

[24] Bertalmio M, Vese L, Sapiro G, Osher S (2003). Simultaneous structure and texture image inpainting. *IEEE Transactions on Image Processing*.

[25] Atzori L, de Natale FGB (1999). Error concealment in video transmission over packet networks by a sketch-based approach. *Signal Processing: Image Communication*.

[26] Rareş A, Reinders MJT, Biemond J (2005). Edge-based image restoration. *IEEE Transactions on Image Processing*.

[27] Hadhoud MM, Moustafa KA, Shenoda SZ Digital images inpainting using modified convolution based method.

[28] Perona P, Malik J (1990). Scale-space and edge detection using anisotropic diffusion. *IEEE Transactions on Pattern Analysis and Machine Intelligence*.

[29] Daisy M, Tschumperlé D, Lézoray O A fast spatial patch blending algorithm for artefact reduction in pattern-based image inpainting.

